# Computed tomography-guided percutaneous neurolysis of celiac plexus: technical description

**DOI:** 10.1590/0100-3984.2019.0005

**Published:** 2020

**Authors:** Renata Motta Grubert, Tiago Kojun Tibana, Larissa Araújo Missirian, Thaline Mairace Hernandez das Neves, Thiago Franchi Nunes

**Affiliations:** 1 Hospital Universitário Maria Aparecida Pedrossian da Universidade Federal de Mato Grosso do Sul (HUMAP-UFMS), Campo Grande, MS, Brazil.

## INTRODUCTION

**Figure 1:**
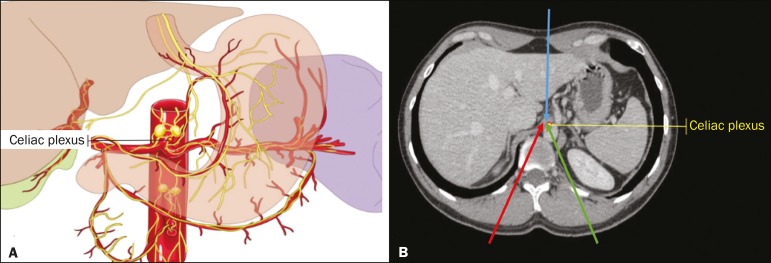
A: Illustration showing the location of the celiac plexus. B: Axial CT scan showing some approaches: anterior hepatic (blue arrow), posterior intervertebral (red arrow), posterior aortic (green arrow).

Abdominal pain is a significant debilitating problem that is common in cancer patients, dramatically affecting quality of life and survival^(1,2)^. The pain, which originates from the viscera of the upper abdomen, is transmitted by visceral afferent fibers that relay the impulses through the splanchnic nerves and the celiac plexus. As illustrated in Figure 1A, the celiac plexus is a network of nerve fibers located in the retroperitoneum along the anterolateral wall of the abdominal aorta^(2,3)^.

The management of cancer-related abdominal pain is complex and challenging, often requiring the chronic use of high doses of opioids, which in turn are generally associated with several adverse effects^(1,2)^. Although therapies employing imaging-guided percutaneous methods reduce the need for potent analgesics and the associated toxicity, such procedures are not widely disseminated and are underused^(1,4-8)^.

Celiac plexus neurolysis (CPN) is a technique that provides permanent interruption of plexus pain transmission through chemical ablation, potentially improving pain control while dramatically reducing opioid consumption^(9,10)^. It involves the infusion of a neurolytic agent, typically sterile absolute alcohol, through a fine needle inserted into the retroperitoneum, adjacent to nerve fibers and the ganglia of the celiac plexus. The neurolytic agent disrupts the neural network, interrupting the pain pathways^(2,11)^. Imaging guidance for CPN is most often performed by computed tomography (CT), which has replaced fluoroscopy and ultrasound for that purpose^(2,12)^.

## PROCEDURE

The first step in CT-guided NPC is pre-procedure planning. Preoperative images should be reviewed in detail to determine the positioning of the patient, as well as to select the puncture site, needle path, and neurolytic injection site (Figure 1B). The pre-procedure planning ensures that the agent is properly distributed, increases the analgesic effect, and reduces morbidity. Proper patient positioning is essential for a successful procedure, because it not only allows a safe percutaneous path to be mapped but also ensures patient comfort.

**Figure 2:**
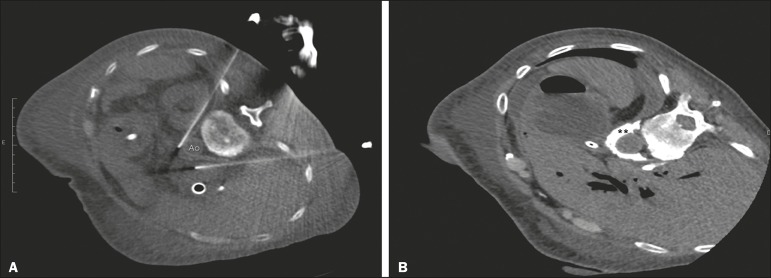
A: Axial CT scan, with the patient in the supine oblique position, showing bilateral paravertebral posterior punctures, adjacent to the aorta (Ao), at the level of the celiac trunk, made with a 22G Chiba needle. B: Correct distribution of absolute alcohol solution and iodinated contrast (asterisks).

Various approaches to CPN can be taken, including anterior and posterior approaches, the bilateral posterior paravertebral approach being the one most frequently employed (Figure 2A). Local anesthesia at the puncture site is performed under sedation with a benzodiazepine or an opioid, and oxygen is delivered via a nasal cannula.

After CT-guided puncture and verification of the correct positioning of the needle(s), with or without injection of 1-2 mL of iodinated contrast agent, a total volume of 40-60 mL of the neurolytic agent (absolut alcohol solution) is infused (Figure 2B). At our facility, we also administer 2-3 mL of 1% lidocaine, without a vasoconstrictor, before and after injection of the alcohol.

As a tool for palliative pain management, CPN is safe and effective, with a relatively low complication rate. It should be offered to patients as a key component of a multidisciplinary approach to the control of intractable chronic abdominal pain. Proper use and knowledge of imaging examinations and of the technique are invaluable to ensuring satisfactory results.
